# Hypertonic Saline as Maintenance Intravenous Fluid in Diabetic Ketoacidosis

**DOI:** 10.1155/crcc/6209006

**Published:** 2026-07-31

**Authors:** Mahalia Sanon, Stanley J. Linder

**Affiliations:** ^1^ Department of Pharmacy, Broward Health Medical Center, Fort Lauderdale, Florida, USA, browardhealth.org; ^2^ Department of Medicine, Broward Health Medical Center, Fort Lauderdale, Florida, USA, browardhealth.org

**Keywords:** brain edema, diabetic ketoacidosis, hyperosmolar hyperglycemic syndrome, hypertonic saline solution, osmolality, sodium

## Abstract

**Introduction:**

Diabetic ketoacidosis is associated with cerebral edema, which, although rare, is associated with an increased mortality rate.

**Case Report:**

In this case report, we discuss the use of a hypertonic saline solution in a 42‐year‐old male with wild‐type gliosarcoma who presented with hyperglycemia to avoid cerebral edema.

## 1. Introduction

Diabetic ketoacidosis (DKA), a complication of diabetes mellitus, is associated with cerebral edema. Although rare, cerebral edema may lead to increased intracranial pressure and herniation and is associated with a 30% mortality rate [1]. Prevention of cerebral edema includes a gradual decrease of serum glucose, sodium, and osmolarity.

## 2. Case Report

A 42‐year‐old male with a past medical history of newly diagnosed wild‐type gliosarcoma presented to the emergency department with altered mental status. The patient was recently hospitalized the month prior and underwent a parietal craniotomy for total resection of the intra‐axial brain tumor during that admission. Home medications included dexamethasone, temozolomide, sulfamethoxazole–trimethoprim, ondansetron, famotidine, metoprolol, cyproheptadine, and levetiracetam. The patient′s family members reported a decline in function since starting chemotherapy, including decreased appetite, polydipsia, polyuria, nausea, and “a burning throat.” His vital signs on presentation included a rectal temperature of 37.2°C, heart rate of 110 bpm, respiratory rate of 24 bpm, and blood pressure of 109/52 mmHg.

On initial physical exam, Glasgow Coma Score was 6. CT brain with and without contrast showed continued evolution of the postsurgical changes related to prior mass resection; however, there is no acute intracranial abnormality. Due to tachypnea and mental status, the patient was intubated in the emergency department. Initial arterial blood gas analysis showed a pH of 7.19, pCO2 of 29 mmHg, and bicarbonate of 12 mEq/L. Labs on presentation included a sodium of 161 mmol/L, potassium of 5.3 mmol/L, chloride of 113 mmol/L, bicarbonate of 9 mmol/L, glucose of 1266 mg/dL, and anion gap of 39. When accounting for hyperglycemia, corrected sodium was 189 mmol/L. Initial lactic acid was 4.9 mmol/L, beta‐hydroxybutyric acid was 11.18 mmol/L, and serum osmolality was 511 mOsm/kg. Calculated osmolality utilizing the measured sodium of 161 mmol/L was 421 mOsm/kg with an osmolar gap of 90 mOsm/kg. The corrected sodium (189 mmol/L) was also used for the calculated osmolality, which resulted in a value of 487 mOsm/kg and an osmolal gap of 24 mOsm/kg. Hemoglobin A1c was 4.8%. Urine analysis was significant for a specific gravity of 1.033, glucose greater than 1000 mg/dL, pH of 5, and ketones of 40 mg/dL.

Due to hypernatremia and hyperglycemia in the setting of dexamethasone use, a mixed DKA and hyperosmolar hyperglycemic state (HHS) was suspected. The patient was placed on an insulin drip with a 1.15% sodium chloride infusion via a central line at a rate of 150 mL/h as maintenance fluid to avoid rapid reduction of serum tonicity resulting in cerebral edema. This concentration was chosen to maintain a corrected sodium of 177–179 mmol/L while treating serum glucose with intravenous regular insulin. The solution was compounded under sterile conditions in the central pharmacy using sodium chloride and sterile water. The rate of the infusion was selected per the institution′s protocol for DKA, and the patient remained on this concentration for 10.5 h, receiving 1575 mL of 1.15% sodium chloride. Once serum blood glucose decreased to less than 250 mg/dL, the intravenous fluid was switched to D5W‐1.15%NS at 150 mL/h. The patient remained on this infusion for 11 h, receiving a total volume of 1650 mL. A CT brain was obtained on Day 2 of hospitalization that did not show any cerebral edema with the measured serum sodium being 176 mmol/L; therefore, maintenance intravenous fluid was switched to D5WNS with 20 mEq of potassium chloride at 150 mL/h.

DKA resolved by hospital day two, as evidenced by an anion gap less than 12 mmol/L and beta hydroxybutyric acid less than 3 mmol/L. The patient remained in the intensive care unit for 8 days and was discharged from the hospital after 24 days. During the hospital stay, the patient developed sinus pauses, which were evaluated by cardiology and attributed to severe electrolyte abnormalities and beta‐blocker use. No other complications occurred during the hospital admission.

## 3. Discussion

This patient was treated with a regular insulin continuous infusion and 1.15% sodium chloride (196 mEq of sodium per liter) as initial maintenance intravenous fluid. Although normal saline (154 mEq of sodium per liter) is the standard initial fluid as part of the institution′s DKA protocol, 1.15% sodium chloride was chosen due to the concern of cerebral edema in the setting of hypernatremia and hyperosmolarity to avoid rapid decreases of serum tonicity while managing the severe hyperglycemia and hypernatremia. Alternative and commercially available hypertonic saline solutions were avoided to achieve a decrease in the corrected sodium by 10–12 mmol/L within 24 h.

Cerebral edema is characterized by increased intracranial pressure causing confusion, seizures, coma, and possibly death from brain herniation. It occurs as a result of a shift in extracellular and intracellular fluids, as well as rapid changes in osmolality. Increased extracellular fluid tonicity leads to the movement of water out of the cellular compartment of the brain [2]. The risk of cerebral edema increases when a rapid decline in extracellular osmolality occurs due to an osmotic shift in the brain.

Cerebral edema may be prevented through careful and gradual replacement of both sodium and water deficits. During hyperglycemic crises, the maximum rate of decline in osmolality is 3–8 mOsm/kg/h and a rapid decline in serum sodium should be limited [3]. The debate between maintaining the corrected sodium at the same level during the early phases of treatment for hyperglycemia in order to limit changes of serum osmolality from both reductions of glucose and sodium or decreasing corrected sodium at a slow rate requires further investigation. Guidelines for management of hyperglycemic emergencies in adults recommend that if cerebral edema is suspected, treatment with mannitol or hypertonic saline should be initiated, even if imaging is not immediately available [4]. During later phases of treatment, a hypotonic infusion is typically required to decrease the corrected sodium [3].

Glucose contributes to tonicity in hyperglycemic states including HHS and water shifts between the intracellular and extracellular compartments based on the change in tonicity [5]. Serum osmolality rapidly decreases as insulin is administered to resolve hyperglycemic crises.

Furthermore, serum tonicity decreases during correction of hyperglycemia without any further changes in body water or monovalent cations [6]. Rather than estimating serum tonicity utilizing serum sodium, corrected sodium predicts the expected serum tonicity after treatment and can be used to guide selection of replacement fluids while normalizing glucose [3, 7]. The corrected sodium, calculated as either a serum sodium increase of 1.6 or 2.4 mEq/L for every 100 mg/dL increase in glucose, is often utilized to evaluate fluid loss through osmotic diuresis in hyperglycemic crises [8, 9].

In one case report, a patient with DKA was erroneously thought to have an elevated osmolal gap of 45 mOsm/kg rather than a normal gap of 7 mOsm/kg because calculated osmolality did not factor in for hyperglycemia [10]. In our patient, serum sodium resulted in an osmolal gap of 90 mOsm/kg compared with a gap of 24 mOsm/kg when using the corrected sodium.

Traditionally, the serum sodium, not corrected, is utilized to calculate the osmolar gap. A comparative study hypothesized that calculated osmolality underestimates true osmolality when concentrations of unmeasured substances such as acetone, amino acids, and glycerol increase and the plasma water fraction decreases [11]. The author found that initial sodium concentrations by indirect potentiometric methods would underestimate plasma sodium, therefore, leading to erroneously low osmolality calculations, and concluded that calculations of osmolality should be modified to include consideration of plasma water content when that content is likely to deviate significantly from normal. On the contrary, Bhagat et al. [12] observed a decreased osmolar gap when calculating plasma osmolality using sodium results obtained by direct potentiometry within 2 h of admission for severe DKA. The authors regarded this difference as artifact due to predilution of samples with a buffered solution. Hence, an alternative strategy in cases of large osmolar gap is to remeasure serum sodium using methods that do not require dilution of the measured specimen to obtain an accurate estimate of the presenting tonicity [4]. This can be accomplished using venous blood gases with electrolytes, which utilize the direct potentiometry method to measure sodium.

In our case, rather than remeasuring the serum sodium, we utilized hypertonic saline with a goal of maintaining a corrected sodium of at least 175 mmol/L to prevent a rapid shift in osmolality. The medical team switched the patient to a dextrose‐containing isotonic solution after the DKA resolved and neurologic examination and brain imaging no longer raised concern for cerebral edema.

Data on the use of hypertonic saline as a maintenance intravenous fluid in DKA are limited, and most case reports are in pediatric patients. The use of hypertonic saline, however, has improved neurologic exam, including Glasgow Coma Score, and imaging [13]. This may be due to an increase in serum osmolality while simultaneously receiving intravenous insulin, therefore, reducing the risk of cerebral edema. Guidelines recommend the use of hyperosmolar agents, such as mannitol and hypertonic saline, if cerebral edema is suspected [4]. Hypertonic saline must be used cautiously and requires frequent monitoring by clinicians. Further studies evaluating the use of hypertonic saline would be necessary; however, maintaining an increased osmolality and serum sodium may reduce the risk of cerebral edema.

## 4. Conclusion

The use of a 1.15% hypertonic solution in an adult patient with concern for cerebral edema in the setting of DKA was safe and efficacious. There may be a role for the cautious use of hypertonic saline in the setting of high osmolality in DKA and HHS; however, further studies are warranted. Further, when calculating osmolality in the setting of extreme hyperglycemia, utilizing the corrected sodium, not serum sodium, should be considered.

## Funding

No funding was received for this manuscript.

## Consent

No written consent has been obtained from the patient as there is no patient identifiable data included in this case report or series.

## Conflicts of Interest

The authors declare no conflicts of interest.

## Data Availability

Research data are not shared.
